# Integrated *in vivo* genetic and pharmacologic screening identifies co-inhibition of EGRF and ROCK as a potential treatment regimen for triple-negative breast cancer

**DOI:** 10.18632/oncotarget.10230

**Published:** 2016-06-22

**Authors:** Sedef Iskit, Cor Lieftink, Pasi Halonen, Aida Shahrabi, Patricia A. Possik, Roderick L. Beijersbergen, Daniel S. Peeper

**Affiliations:** ^1^ Department of Molecular Oncology, The Netherlands Cancer Institute, Plesmanlaan, Amsterdam, The Netherlands; ^2^ Department of Molecular Carcinogenesis, The Netherlands Cancer Institute, Plesmanlaan, Amsterdam, The Netherlands; ^3^ Drug Discovery Research and Screening Services, BioFocus, Darwinweg, Leiden; ^4^ Brazilian National Cancer Institute, Rio de Janeiro, Brazil

**Keywords:** in vivo screen, targeted therapy, TNBC, EGFR, ROCK

## Abstract

Breast cancer is the second most common cause of cancer-related deaths worldwide among women. Despite several therapeutic options, 15% of breast cancer patients succumb to the disease owing to tumor relapse and acquired therapy resistance. Particularly in triple-negative breast cancer (TNBC), developing effective treatments remains challenging owing to the lack of a common vulnerability that can be exploited by targeted approaches. We have previously shown that tumor cells have different requirements for growth *in vivo* than *in vitro*. Therefore, to discover novel drug targets for TNBC, we performed parallel *in vivo* and *in vitro* genetic shRNA dropout screens. We identified several potential drug targets that were required for tumor growth *in vivo* to a greater extent than *in vitro*. By combining pharmacologic inhibitors acting on a subset of these candidates, we identified a synergistic interaction between EGFR and ROCK inhibitors. This combination effectively reduced TNBC cell growth by inducing cell cycle arrest. These results illustrate the power of *in vivo* genetic screens and warrant further validation of EGFR and ROCK as combined pharmacologic targets for breast cancer.

## INTRODUCTION

Breast cancer is the most commonly diagnosed cancer and the second most common cause of cancer-related deaths among women worldwide [1]. Although several treatment options are available, 15% of breast cancer patients succumb eventually, due to relapse and/or distant metastases that are associated with acquired resistance to current therapies. Especially triple negative breast cancer (TNBC), which is named so due to the absence of ER, PR and HER2 receptors and constitute 15-20% of all breast cancers, has proven to be challenging owing to the lack of a common vulnerability that can be exploited by targeted approaches.

Although tumors generally have a high number of mutations, genomic instability and chromosomal aberrations [2], only a fraction of these events contributes to tumor formation and progression [3]. In the last decade, there have been several sequencing efforts to unfold the mutational landscape of tumors, to identify driver mutations and develop targeted therapies [3–7]. In the case of breast cancer, targeted endocrine therapies can be used for ER+ and HER2+ tumors, but are ineffective for the triple negative subtype due to the absence of expression of hormone receptors. In fact, TNBC lacks such oncogene signatures that would allow tumors to be sub-grouped and specifically targeted. Aside from p53 mutations (80%), the most common genetic aberration in TNBC is in PI3K, which occurs in 9% of the cases [6]. Additionally, a set of TNBC displays targetable BRCA-like clinical and pathological features, which render these tumors sensitive to PARP inhibition [8]. More recently, a detailed analysis of somatic alterations in breast cancer samples revealed a number of mutations that are specific to the basal-like breast cancer subtype [9]. However, a significant proportion of patients are still dependent on chemotherapeutic approaches.

Although the majority of TNBC tumors initially respond to conventional chemotherapy, TNBC patients still have a poor prognosis [10, 11]. Numerous clinical studies have been conducted in recent years to assess the effectiveness of targeted agents such as PARP, EGFR and VEGF inhibitors in different settings. These efforts, however, either failed to significantly improve patient survival or were inconclusive [12, 13]. It therefore is crucial and urgent to identify novel therapeutic targets to treat TNBC.

At least as important as oncogene addiction is the concept of non-oncogene addiction or essentiality, reflecting that genetically unaltered and normally functioning pathways (which would not be identified by sequencing) can also be critical to a tumor's maintenance [14]. This phenomenon greatly expands the spectrum of potential drug targets for cancer treatment. In line with this, and because TNBC lacks genetically obvious common vulnerabilities, we sought to identify novel drug targets by performing unbiased genetic screening, which is a powerful tool that has been widely employed in cancer research to search for novel targets for therapy [15]. Since *in vitro* screens typically fail to cover crucial components that contribute to tumor progression such as stromal interactions, immune system, and vascular structure, *in vivo* screens have become a more favorable approach [16, 17]. We recently uncovered a synthetic lethal effect of hypoxia and DNA damage response inhibition by a similar approach [18], illustrating the power of performing such screens in an *in vivo* setting. Therefore, we set out to carry out parallel *in vivo* and *in vitro* loss-of-function shRNA screens for the identification of novel targets for breast cancer. Identified targets were subsequently interrogated with pharmacological inhibitors using combination screens to identify effective, synergistic combinations.

## RESULTS

### Screening for kinases that are required for tumor growth *in vivo*

**Figure 1:**
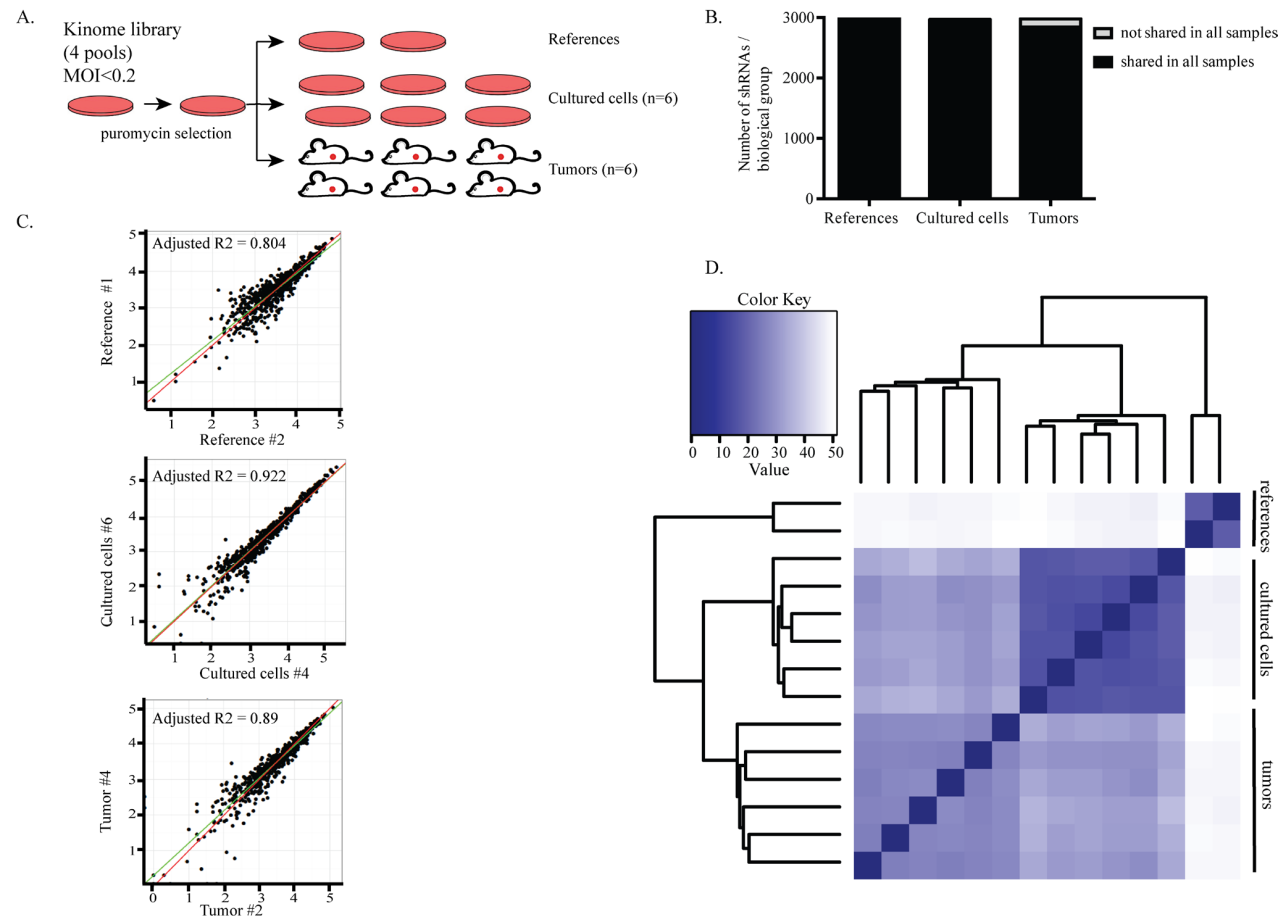
Screening for kinases that are required for tumor growth *in vivo* **A.** Set-up of the screens. HCC1806 and MDA231 cells were transduced with the kinome library in four pools with lentivirus at an MOI<0.2. After three days of puromycin selection, reference samples were collected and the remainder of the cells were either transplanted into the mammary fat pads of six NSG mice or seeded into six independent dishes for the *in vitro* screen. **B.** The complexity of the library was retained among all groups in the HCC1806 cell screen. Bars show the average number of shRNAs per biological group. Of the 2997 shRNAs detected in the reference samples, 2882 and 2710 were also found in cultured cells and tumors, respectively. Dark parts of the bars represent the shared shRNAs among the biological replicates within a group. 96% of the shRNAs were commonly found among the cultured cells while 90% were common among the tumors. **C.** Biological replicates correlated well with each other. A representative example from each sample group is shown. Every dot represents an shRNA. X- and y-axis show the abundance of shRNAs. **D.** Euclidean distance heat map showing the degree of similarity between all samples. All biological replicates in a sample group cluster together.

In order to establish a physiologically relevant model and identify more clinically realistic targets, we performed an *in vivo* screen with a parallel *in vitro* counterpart. This system allowed us to specifically uncover those genes that are more critical for tumor survival *in vivo* compared to *in vitro* [18]. Because tumors highly rely on kinase pathways and new therapies targeting kinases are being widely explored [23], we chose to use a kinome library derived from the genome-wide TRC library [24] and composed of ~3000 shRNAs targeting ~500 kinases [18, 25]. Two TNBC cell lines, HCC1806 and MDA-MB-231, were transduced with the kinome library in four pools (Figure 1A). After three days of antibiotic selection for successful transduction and expansion, reference samples were collected. The remaining cells were either injected into the mammary fat pads of six NSG mice (*in vivo* screen) or seeded in tissue culture dishes in six replicates (*in vitro* screen). Tumors were harvested once they reached 50-100mm^3^ and the cultured cells were harvested after two expansions. The presence of each shRNA in reference, *in vitro* and *in vivo* samples was quantified using genomic DNA extraction followed by PCR amplification and deep sequencing.

Before hit calling, we performed several quality control analyses to confirm that the data generated from the screens was sufficiently robust for negative selection analyses. First, quantification of the shRNAs present in tumors and in *in vitro* samples showed that the complexity of the library was maintained throughout the experiment, as we could detect approximately 3000 unique shRNAs in the references, cultured cells and tumor samples. Importantly, the majority of these shRNAs were shared amongst all sample groups. Specifically, 85% were shared between the cultured cells and tumors. These findings indicate that the complexity of the library was well maintained; this allowed the identification of shRNAs that were lost due to functional selection of a specific shRNA rather than random selection of shRNAs as a result of sampling due to clonal expansion (Figure 1B, Supplementary Figure 1A).

We observed a high correlation of shRNAs between biological replicates (Figure 1C, Supplementary Figure 1B). Unsupervised clustering analysis showed that, for each experimental group, all biological replicates clustered into one branch, suggesting that the abundance of shRNAs present in these replicates is reproducible and supporting the robustness of the system (Figure 1D, Supplementary Figure 1C).

### Identification of *in vivo*-specific targets

**Figure 2:**
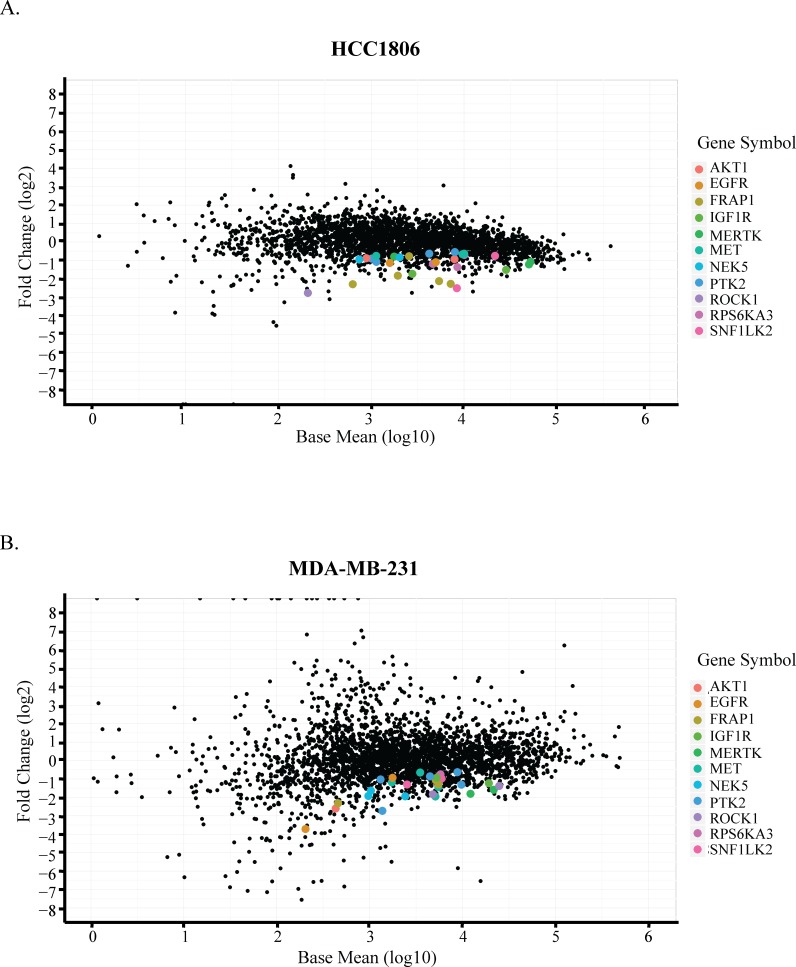
Identification of *in vivo*-specific targets Comparison of tumors to cultured cells by DESeq analysis in **A.** HCC1806 and **B.** MDA-MB-231 cells. X-axis shows the average abundance of each shRNA across all samples on log_10_ scale. Y-axis shows the fold change of each hairpin in tumors compared to the cultured cells in log_2_ scale. Common hits identified based on the criteria are color-coded.

*In vivo*-specific hits were identified based on the following criteria: 1) an shRNA should be significantly depleted (*p* < 0.01) and have an effect size of at least 30% in tumors compared to *in vitro* samples; 2) a gene should be represented with at least two shRNAs in the screen; 3) an shRNA for a selected gene in (2) should not be enriched more than 20% in *in vitro* samples compared to the references; and 4) an shRNA for a selected gene in (2) should not be enriched in tumor samples compared to the references. For the genes targeted by shRNAs fulfilling these criteria, we compared the hit lists from both HCT1806 and MDA-MB-231 screens to finally generate a list composed of genes identified in both screens, corresponding to the fifth selection criterion (Figure 2, Table 1). The hit list comprised receptor tyrosine kinases (EGFR, MERTK, IGF1R), intracellular signal transducers (AKT1, MET, mTOR, RSK2), cytoskeletal regulators (FAK, ROCK1), and some functionally under-investigated genes (NEK5, SIK2).

**Table 1:** Common hits from the two *in vivo* screens

	number of shRNAs		
	MDA-MB-231	HCC1806	common
**AKT1**	2	2	0
**EGFR**	2	3	1
**mTOR**	2	5	2
**IGF1R**	2	2	1
**MERTK**	2	2	1
**MET**	4	3	1
**NEK5**	3	2	2
**FAK**	5	4	1
**ROCK1**	2	2	0
**RSK2**	2	2	1
**SIK2**	2	2	2

Columns indicate how many shRNAs for the corresponding gene were found in each screen and how many of these were the exact same shRNA.

### Uncovering synergistic combinations by pharmacologically targeting hits

By using the kinome library for our screens, we wished to take advantage of the fact that kinases are relatively easy to target pharmacologically, allowing us to translate our results to a pre-clinical setting. Notably, previous experiences on targeted therapy approaches have shown that most single-agent treatments fail to offer a long-term solution as tumors commonly recur because of drug resistance [26]. Moreover, an effective combination of two or more targeted agents in TNBC is lacking. We therefore sought to identify synergistic combinations between our hits in both MDA-MB-231 and HCC1806 cells using an *in vitro* drug matrix setting. It is important to point out here that the *in vivo*-*in vitro* differences observed are not absolute but rather reflect sliding windows. Therefore, certainly when combining the inhibition of multiple targets, we expected to see significant effects in *in vitro* assays also, aiming to find potential new treatment regimens.

We selected the set of genes from our hit list against which commercially inhibitors are available that are either already FDA-approved or are being evaluated in clinical trials for different types of cancer and other diseases. These were combined with each other in 5×5 dose matrices. Establishing single-treatment dose response curves with each matrix allowed us to compare combined treatments with the expected additive effects of singe treatments (Supplementary Figure 2). The results are presented as synergy scores after calculating the differences between the expected and the actual effects (Table 2a–2b). Scores >1 are considered synergistic, provided that the self-self combination of each drug has a lower score than the combination score.

**Table 2a:** Synergy scores of combination treatments in HCC1806 cells

HCC1806								
TARGETS	ROCK	EGFR	mTOR (1)	mTOR (2)	AKT	FAK	FGFR	MET
**ROCK**	0.74	**4.25**	0.82	0.16	0.97	0.13	4.61	1.01
**EGFR**		0.58	0.93	2.38	1.91	2.25	0.92	0.19
**mTOR (1)**			0.74	2.24	**7.63**	0.67	0.28	1.7
**mTOR (2)**				1.97	**2.51**	2.05	0.11	4.16
**AKT**					1.16	0.23	0.14	0.16
**FAK**						0.18	0	0.93
**FGFR**							0.3	1.54
**MET**								1.59
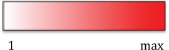								

The synergy scores of all combination treatments, including self-self combinations are shown. Because scores >1 was considered synergistic, the heat map grades scores between 1 and the maximum score in the table.

**Table 2b:** Synergy scores of combination treatments in MDA-MB-231 cells

MDA231								
TARGETS	ROCK	EGFR	mTOR (1)	mTOR (2)	AKT	FAK	FGFR	MET
**ROCK**	0.33	**3.16**	0.63	0.5	0.71	0	0.73	0
**EGFR**		0.93	0.29	0.14	0.1	0	0.11	1.12
**mTOR (1)**			0.12	1.37	**2.97**	0.26	0.14	0.07
**mTOR (2)**				0.99	**1.7**	0.66	0.42	0.69
**AKT**					0.33	0.46	0.42	0.13
**FAK**						0.18	0.13	0.02
**FGFR**							0.63	0.01
**MET**								0.83
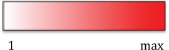								

The synergy scores of all combination treatments, including self-self combinations are shown. Because scores >1 was considered synergistic, the heat map grades scores between 1 and the maximum score in the table

To rule out cell line-specific effects, we selected combinations that showed synergistic effects in both TNBC cell lines tested. One such combination, two mTOR inhibitors (AZD8055 and Everolimus), was excluded from the analysis because both target the same kinase. Within the same pathway, we observed synergy when combining AZD8055 or Everolimus with the AKT inhibitor MK2206. This finding was reassuring, since synergistic effects of AKT and mTOR inhibition have previously been reported for other cancer types [27–30]. With this experimental approach we observed a highly synergistic effect when combining the EGFR inhibitor Gefitinib (EGFRi) with the ROCK inhibitor GSK269962A (ROCKi) (Supplementary Figure 3). Based on these results, and on the results of the shRNA screen on the same cell lines, we focused on this combination for further investigation of potential therapeutic applications in TNBC.

### EGFR and ROCK1 depletion impairs TNBC growth

**Figure 3:**
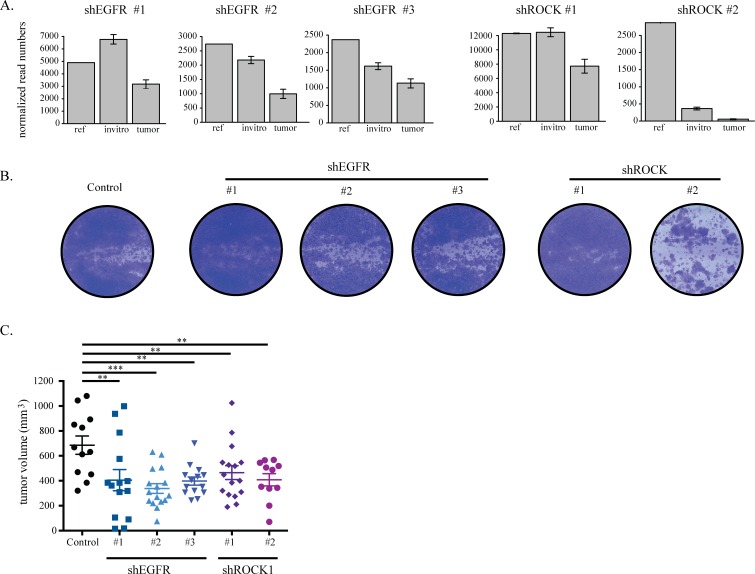
EGFR and ROCK1 depletion impairs TNBC growth **A.** shRNA read counts of the hairpins in the reference, cultured cells (invitro) and tumor samples as found in the HCC1806 screen. **B.** HCC1806 cells were transduced with lentiviral constructs against EGFR and ROCK that were identified as hits in the screen. After puromycin selection, 0.3*10^5^ cells were seeded on 6-well plates. Cells were stained with crystal violet after six days. **C.** HCC1806 cells transduced with lentiviral constructs against luciferase, EGFR and ROCK were orthotopically injected into the 4^th^ mammary fad pad of NSG mice. Tumors were measured manually by a caliper and tumor volume was calculated by the formula a*b^2^/2.

We confirmed that the different shRNAs against EGFR and ROCK1 that scored as hits in the screens reproducibly caused effects similar to those seen in the screen. The identification of multiple unique shRNAs for both EGFR and ROCK1 rules out a possible off-target effect. To compare this validation with the screening results, we studied in more detail the shRNA distribution in each experimental group of the *in vitro* and *in vivo* screens. As expected, we observed a more pronounced loss of shRNAs targeting EGFR and ROCK1 in tumors as compared to both the references and the *in vitro* cultured cells (Figure 3A, Supplementary Figure 3A). We then transduced TNBC cell lines with the individual shRNAs against EGFR and ROCK1 and showed efficient silencing of the target genes (Supplementary Figure 3B). Finally, we evaluated the effect of EGFR and ROCK silencing on tumor cell viability *in vitro* and growth *in vivo*. *In vitro,* cell proliferation followed the same pattern predicted by the screen results: knock down of either gene showed little effect on the cell lines, with the exception of hairpin #2 targeting ROCK1 in HCC1806 cells (Figure 3B, Supplementary Figure 3C). As all hairpins for ROCK1 successfully depleted the protein, we could not determine whether this reflected a difference in remaining ROCK1 levels (Supplementary Figure 3B). *In vivo*, on the contrary, silencing of either ROCK1 or EGFR impaired growth of HCC1806 xenografts (Figure 3C).

### Combined EGFR and ROCK inhibition effectively blocks proliferation of TNBC cells

**Figure 4:**
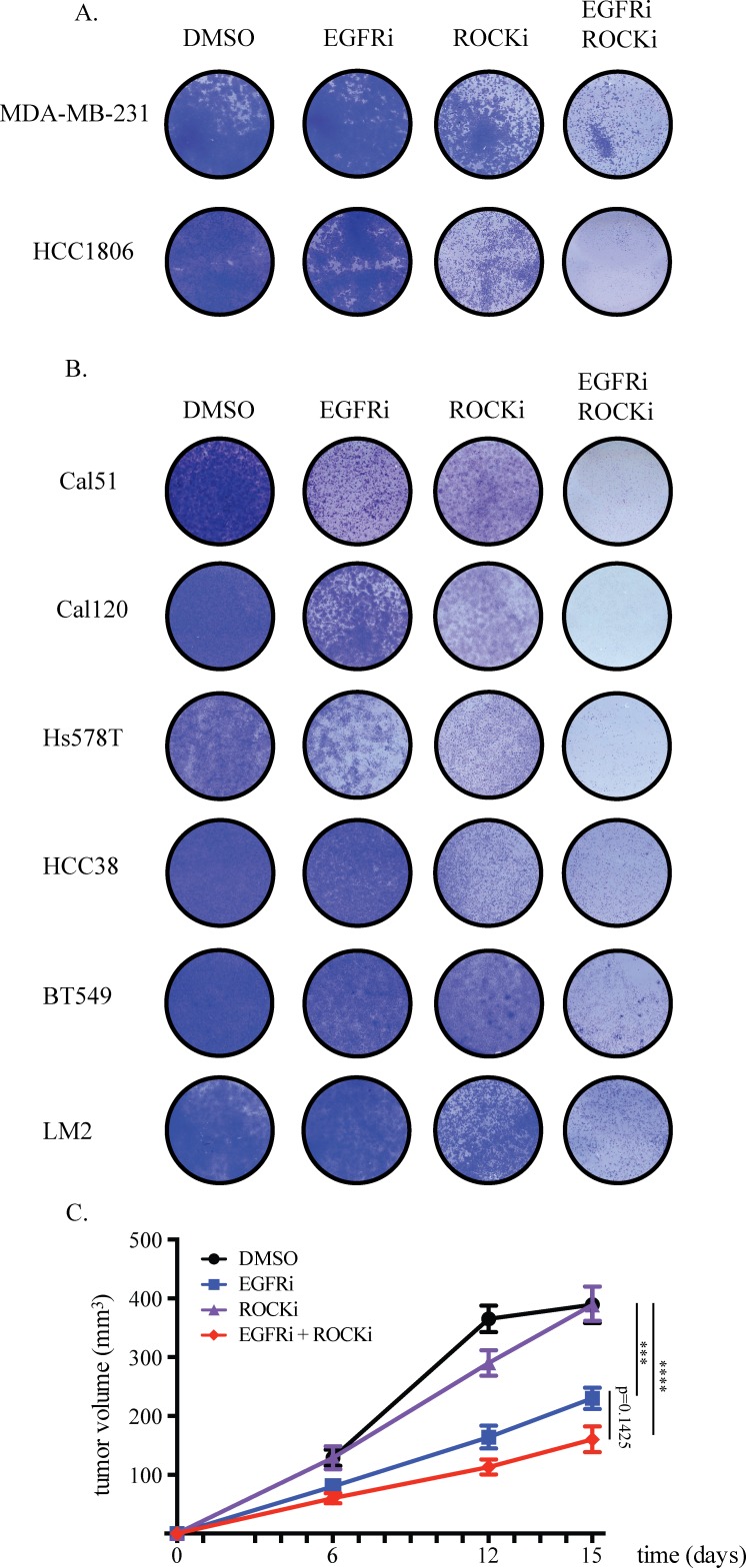
Combined EGFR and ROCK inhibition effectively blocks proliferation of TNBC cells **A.** 0.1*10^5^ cells were seeded onto 12-well plates. Drugs were added one day after seeding and refreshed at the third day. MDA-MB-231 cells were treated with 20μM Gefitinib (EGFRi), 4.8μM GSK269962A (ROCKi) or the combination. HCC1806 cells were treated with 8.4μM EGFRi, 2.4μM ROCKi or the combination. Same doses were used for the combination treatments. Cells were stained with crystal violet six days after treatment. **B.** EGFRi-ROCKi combination has enhanced toxicity also on other TNBC cell lines. EGFRi concentration for Cal51, Cal120, Hs578T, HCC38, BT549 and LM2 cells was 20μM. ROCKi concentration for Cal51 cells was 12μM, for Cal120 cells was 30μM, for Hs578T cells 1.2μM, for HCC38 cells 12μM, for BT549 cells 8μM, for LM2 cells 4.8μM. **C.** HCC1806 cells were orthotopically injected into the 4^th^ mammary fat pad of NSG mice. Starting from one day after inoculation, mice were orally treated six times a week with DMSO-containing vehicle, 90mg/kg EGFRi, 10mg/kg ROCKi, or EGFRi+ROCKi. Tumors were measured twice weekly and the tumor volumes were calculated by the formula a*b^2^/2.

Because the synergistic effect of EGFR and ROCK inhibitor combination was identified in 72-hour dose-response assays, we performed longer-term experiments and confirmed that this combination has a major impact on the expansion of both MDA-MB-231 and HCC1806 cells while single inhibitor treatment only mildly impairs growth (Figure 4A). The effect of the inhibitors on EGFR and ROCK signaling was confirmed by analyzing EGFR, AKT and MYPT phosphorylation status upon EGFR and ROCK inhibitor treatments, respectively (Supplementary Figure 4A). In addition, we found similar results with other inhibitors of EGFR and ROCK, Afatinib and Fasudil, respectively, suggesting that this is indeed a target-specific and not a compound-specific effect (Supplementary Figure 4B). Moreover, double knockdown of EGFR and ROCK impaired the growth of HCC1806 cells, further supporting specificity of the effect of the inhibitors combination (Supplementary Figure 4C).

The growth inhibition caused by EGFRi and ROCKi combination was not limited to MDA-MB-231 and HCC1806 cells. We observed substantial growth impairment in a panel of another six TNBC cell lines also (Figure 4B). Furthermore, compared to each inhibitor alone, the inhibitory growth effect of the combination on orthotopic tumors formed by HCC1806 cells was enhanced, however did not reach statistical significance (Figure 4C). The combinatorial effect of EGFR and ROCK inhibition was independent of the expression levels of EGFR and ROCK proteins or of the phospho-EGFR or phospho-MYPT (downstream target of ROCK) levels (Supplementary Figure 4D). This proof-of-concept result warrants further validation and optimization of the combined pharmacologic targeting of EGFR and ROCK for TNBC.

### Combined EGFR and ROCK inhibition causes cell cycle arrest

We next sought to identify the mechanism by which combined inhibition of EGFR and ROCK impairs cell growth. Consistent with the involvement of ROCK in the regulation of cell shape and movement [31], we observed major changes in cell morphology upon ROCK inhibitor treatment (Supplementary Figure 5A). Cells became flattened, larger and had several protrusions. When EGFR and ROCK inhibitors were combined, the remaining cells acquired neuron-like long extensions.

**Figure 5:**
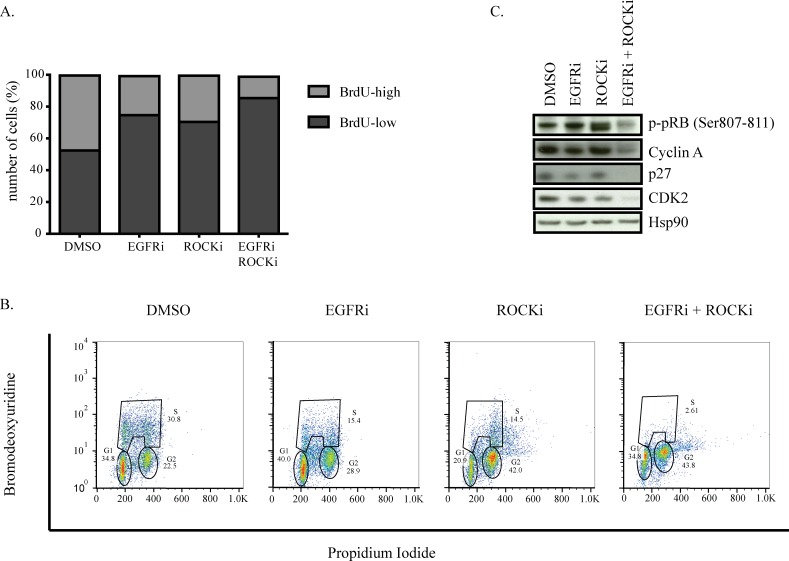
Combined EGFR and ROCK inhibition causes cell cycle arrest **A.** MDA-MB-231 cells were treated with DMSO, EGFRi, ROCKi or EGFRi+ROCKi for two days. Before harvesting, cells were incubated with BrdU for 1.5 hours. Ethanol-fixed cells were stained with anti-BrdU antibody followed by FITC-labeled secondary antibody and analyzed by FACS. **B.** Western blots of MDA-MB-231 cells after 2 days of DMSO, EGFRi, ROCKi or EGFRi+ROCKi treatment. **C.** Cell cycle profile of MDA-MB-231 detected by BrdU-PI co-staining. Tails to the right of G2- and S-phase gates denote polyploid cells.

Since we found very few cells surviving the combination treatment, we investigated whether co-inhibition of EGFR and ROCK causes cell death. However, we did not find any indication of apoptosis such as floating cells, PARP or caspase 3 cleavage, or Annexin V and PI positivity in HCC1806 or MDA-MB-231 cells (data not shown). We therefore investigated whether either of the inhibitor treatments or the combination would affect how these tumor cells progress through the cell cycle. DNA replication, as revealed by BrdU incorporation during the S-phase, was only mildly decreased by the single treatments, whereas the combination of EGFR and ROCK inhibitors completely prevented MDA-MB-231 cells from progressing through this cell cycle phase (Figure 5A). Upon single treatment, we observed a two-fold reduction in the number cells that incorporated BrdU (corresponding to S-phase cells) while combination treatment caused a four-fold reduction.

We next analyzed the individual cell cycle phases of single diploid cells. Consistent with the BrdU incorporation results of the general population, we observed a two-fold reduction in the number of cells that went through the S-phase upon EGFR or ROCK inhibitor treatment. Additionally, ROCK inhibitor alone and in combination with EGRF inhibition caused a two-fold increase in the number of cells in G2 phase as well as an increase in the number of polyploid cells (Figure 5B). Importantly, the number of diploid cells going through S-phase upon combination treatment was almost six times less than singe-drug-treated cells.

Consistently, phosphorylation of pRb, which is essential for the cells to progress from G1 to S phase, was not affected in cells treated with either inhibitor alone but was almost abolished upon combination treatment. The reduction in pRb phosphorylation was accompanied by loss of Cyclin A and Cdk2, as well as p27^Kip1^, which has an essential role in assembling Cyclin-Cdk complexes [58] (Figure 5C). These data suggest that combined inhibition of EGFR and ROCK impedes cells from progressing through the cell cycle, arresting cells in both G1 and G2 phases of the cell cycle and consequently restricting the proliferative capacity of TNBC cells.

## DISCUSSION

Targeted therapy has arisen as an alternative to cytotoxic drugs in cancer therapy, in some cases increasing the response rate of patients as well as overall survival and progression-free survival [32]. The major challenge of targeted therapy is the issue of drug resistance, which eventually develops. More durable clinical responses are seen when targeted therapies were combined with radiotherapy, chemotherapy or other targeted compounds [33, 34]. Here, we report combined EGFR-ROCK inhibition as a potential combination treatment for triple-negative breast cancer.

We performed two loss-of-function screens *in vivo* and *in vitro* using two distinct TNBC cell lines in order to uncover common potential targets in TNBC. We ensured focusing on more clinically relevant targets by 1) using a library comprising shRNAs targeting kinases which are generally targetable by drugs; and 2) comparing shRNA loss in tumors relative to cultured cells, corresponding to the two arms in the screen. Our hit list contains genes coding kinases with established oncogenic functions such as MET [35], EGFR [36, 37], AKT, mTOR [38–40], RSK2 [41] as well as genes that do not (yet) have an established role in cancer progression (NEK5, SIK2). Our hit list also comprised genes like ROCK1 and FAK that are known to play a role in migration, invasion and metastasis but have only recently been implicated in tumor progression [42–45].

It is increasingly clear that for durable clinical responses to occur one needs to develop effective combinatorial strategies [22, 46]. Moreover, unlike genetic manipulation by shRNAs, pharmacologic inhibition of targets allows flexibility in timing, dosing and order of treatment. For these reasons, we combined our *in vivo* screening with an i*n vitro* pharmacologic approach in order to find more effective, combined treatment options. We combined eight compounds with each other in a matrix setting in which five doses of one drug were combined with five doses of a second drug. We used this experimental system to assess the effect on the proliferation of HCC1806 and MDA-MB-231 cells. This resulted in the identification of two pairs of inhibitors that showed synergistic effects: EGFR + ROCK, and AKT + mTOR inhibitors. Since mTOR inhibition increases AKT activity by disrupting feedback inhibition [47, 48], vertical targeting of the PI3K-AKT-mTOR pathway has proven to be a promising approach in several cancer types [27–29], with clinical trials ongoing [30]. We find this combination with two different inhibitors targeting mTOR (Everolimus and AZD8055), validating the accuracy of our system.

The anti-proliferative effect of the combination of a ROCK and EGFR inhibitor on the other hand, has not been described before. ROCK, an effector of the small GTPase Rho, is widely studied in the context of cell shape and movement. It is known to be a major regulator of migration, influencing cellular characteristics such as contraction, actin organization and polarity [49]. Consistent with this, we observed major morphological changes upon ROCK inhibition in all cell lines we analyzed. Aside from its critical role in migration, ROCK inhibition has been shown to cause defects in cytokinesis [50]. In line with this, we observed an increased number of G2 and polyploid cells upon treating with ROCK inhibitor. However, although this slowed down the proliferation rate, it did not severely impair cell growth in the long run. Also *in vivo*, ROCK inhibitor had no impact on tumor growth.

ROCK inhibition as an anti-tumor treatment is not widely investigated but its potential use in combination with chemotherapy or other tyrosine kinase inhibitors is increasingly recognized [51]. For instance, ROCK and Brc-Abl co-inhibition leads to apoptosis and cell cycle defects in chronic myeloid leukemia cells [52]. We also recently showed that in combination with either BRAF, ERK or MEK inhibition, ROCK inhibition delays melanoma growth [25, 53]. In several TNBC cell lines, we observed substantial growth impairment *in vitro* upon treatment with both EGFR and ROCK inhibitors compared with single-agent treatment. A similar pattern was seen for *in vivo* growth of HCC1806 cells. However, unlike the recent findings for lung cancer [54], we did not find a correlation between the response of TNBC cells to combination treatment and EGFR mutation status, phospho-EGFR, phospho-MYPT or ROCK protein levels, suggesting a cell type-specific component.

To begin to explore the mechanistic basis for our observations, we found that co-inhibition of EGFR and ROCK induces cell cycle arrest in HCC1806 and MDA-MB-231 cells. This was accompanied by a decrease in the protein levels of cyclin A, Cdk2, p27 and phosphorylated pRB. Cyclin A - Cdk2 complex is active at two points in the cell cycle: during onset of S phase and in early G2 phase. The function of Cyclin A - Cdk2 in G2 is thought to be the regulation of Cyclin B - Cdk1 levels to enter mitosis [55–57]. Therefore, absence of an active Cyclin A - Cdk2 complex would cause insufficient levels of Cyclin B - Cdk1 complex formation, leading to a failure in mitosis entry and causing the cells to accumulate at G2 phase. Although p27 is known to act as a cell cycle inhibitor by blocking the activity of Cyclin E - Cdk2 complex, it also aids in the assembly of Cyclin D - Cdk4 complexes [58, 59], allowing cells to progress through the G1 phase. Another major inhibitor of the cell cycle is pRB. In its unphosphorylated form, pRB binds to E2F and inhibits its transcriptional activity whereas phosphorylated pRB dissociates from E2F, allowing cell cycle gene expression and entry into the S phase [60]. Co-inhibition of EGFR and ROCK in triple-negative breast cancer cells caused decrease in phosphorylated pRB, p27, Cyclin A and Cdk2 protein levels, correlating with an almost complete loss of the ability of the cells to replicate and consequently, proliferate.

In conclusion, we demonstrate here that EGFR and ROCK are potential *in vivo* targets for triple-negative breast cancer in a combination setting: co-inhibition of EGFR and ROCK has a profound inhibitory impact on cell proliferation in a panel of TNBC cell lines, *in vitro* as well as in mice, which was at least partially explained by the induction of cell cycle arrest. This proof of concept warrants further validation and optimization to determine whether this ought to be studied in the clinic.

## MATERIALS AND METHODS

### Cell culture and reagents

Hek279T, MDA-MB-231, LM2, BT549, Cal51, Cal120 cells were maintained in Dulbecco's modified Eagle's medium (DMEM) supplemented with 9% fetal bovine serum (Sigma), 2mM glutamine, 0.1mg/ml penicillin and 0.1ml/ml streptomycin (Gibco). HCC1806, HCC38, Hs578T cells were maintained in RPMI supplemented with glutamine.

Hek293T cells were used for lentivirus production as described previously [19]. shRNAs targeting EGFR and ROCK were obtained from the TRCs1.0 library and were as follows: shEGFR-1: TRCN0000121068, shEGFR-2: TRCN0000010329, shEGFR-3: TRCN0000121206, shEGFR-4: TRCN0000121203, shROCK1-1: TRCN0000002163, shROCK1-2: TRCN0000121316, shROCK1-3: TRCN0000121095, shROCK1-4: TRCN0000002161 (TRC Library, Sigma).

For long-term cell growth assays, cells were seeded on 6-well or 12-well plates (Corning). Drugs were added on the following day and media was refreshed every third day with new compound dilutions. At the end time point, the cells were stained with crystal violet. ROCK inhibitors used were GSK269962A (Axon) and Fasudil (Selleck). EGFR inhibitors used were Gefitinib (MedChem) and Afatinib (Selleck).

For DNA content and cell cycle analysis, sub-confluent cells were incubated with 10uM Bromdeoxyuridine (BrdU) for 1.5 hours, trypsinized, fixed in 70% ice-cold ethanol, and stained with anti-BrdU and Propidium Iodide (PI).

### *In vivo* and *in vitro* screens

A lentivirus-based Kinome shRNA library targeting ~500 kinases and kinase-related genes with ~3000 shRNAs was assembled from the human genome-wide shRNA collection (TRCHs1.0). The screens were set up and performed as described before [18]. Briefly, 1.5×10^6^ cells were seeded in 10cm culture dishes and infected for 6 hours (MOI<0.2) with lentivirus-containing supernatant. After 3 days of puromycin selection (1 mg/ml), the reference samples were collected. The remaining cells were either mixed with 1:1 matrigel and injected into the 4^th^ mammary fat pad of 6 female NOD/SCID IL2γ^null^ (NSG) mice (0.5×10^6^ cells/mouse), or seeded on 6×10cm dishes and maintained in culture in parallel (0.5×10^6^ cells/dish). The cells and the tumors were harvested after two and three weeks, respectively. This procedure was repeated for each of the 4 Kinome library pools. For the quantification of shRNAs in all samples, gDNA was isolated (DNeasy Blood and Tissue kit, Qiagen) and shRNAs were quantified after PCR amplification and deep sequencing (Illumina HiSeq2000). Results were analyzed with the DESeq package of R/Bioconductor [20, 21]. shRNAs that are detected with less than 200 reads on average in the references and in *in vitro* samples were excluded from the analysis. Normalized read numbers were compared between tumors and cultured cells in order to determine the shRNAs that were lost 30% more *in vivo* than *in vitro*. Genes targeted with at least two shRNAs with a false discovery rate < 0.1 were considered hits, provided that they were not enhanced more than 20% in *in vitro* samples compared with the reference samples.

### Synergy matrix

HCC1806 and MDA-MB-231 cells were seeded onto 384-well plates at 10^3^ and 2.5*10^3^ cells/well, respectively, and treated with 5 serial dilutions of one drug combined with 5 serial dilutions of a second drug in a matrix format. The maximum dose used per drug did not exceed its IC50. In order to obtain a dose-response curve from the drugs in the matrices, cells were treated with six more serial dilutions of higher doses of each drug outside of the matrix. After 72 hours, cells were incubated for two hours in CellTiter Blue at 1:20 dilution and the absorbance was measured at TECAN. Synergy matrix calculations were done as described before [22].

### Immunoblot analysis and antibodies

Cells were harvested in ice by scraping in ice cold 1X PBS and the pellets were lysed in RIPA buffer (50 mM TRIS pH 8.0, 150 mM NaCl, 1% Nonidet P40, 0.5% sodium deoxycholate, 0.1% SDS, complete protease inhibitor cocktail (Roche), and phosphatase inhibitors 10 mM NaF, 1 mM Na_3_VO_4_, 1 mM sodium pyrophosphate, 10 mM beta-glycerophosphate). After sonication and centrifugation the protein concentrations were determined using the Bio-Rad protein assay (Bio-Rad). Samples were loaded on 4-12% Bis-Tris polyacrylamide-SDS gels (NuPAGE) and transferred on to nitrocellulose membranes (Amersham). Membranes were blocked in 4% skimmed milk powder dissolved in 0,2% Tween-containing 1X PBS and incubated with primary antibodies followed by secondary antibodies (Invitrogen). Primary antibodies used were EGFR (sc-03, Santa Cruz), EGFR_Y2068_ (ab5644, Abcam), ROCK1 (611137, BD), MYPT_Thr696_ (ABS45, Millipore), ERK_Thr202/Tyr204_ (4370S, Cell Signaling), ERK (9102, Cell Signaling), Hsp90 (sc-7947, Santa Cruz), p27 (610241, BD), pRB_Ser807/811_ (9308S, Cell Signaling), CDK2 (sc-163, Santa Cruz), Cyclin A (sc-596, Santa Cruz).

### *In vivo* experiments

All animal work was done in accordance with a protocol approved by the Netherlands Cancer Institute Animal Experiment Ethics Committee. Female NSG mice aged 5-8 weeks were used for all *in vivo* experiments. Human breast cancer cells were prepared as 10^7^ cells/ml suspension in medium, mixed 1:1 with growth factor reduced matrigel and 100ul of the mixture was injected into the 4^th^ mammary fat pad of the mice on both sides (0.5*10^6^ cells/injection). Mice were orally treated with drugs 6 days/week, starting one day after tumor inoculation. GSK269962 was dissolved in DMSO at 100mM and diluted in 10% Tween80 and 6.5% ethanol mix to 10mg/kg. Gefitinib was dissolved in DMSO at 200mM and diluted in 2% Tween80 to 90mg/kg. Tumors were manually measured twice a week with a caliper and tumor volume was calculated by the formula a*b^2^/2 where ‘a’ is the longest diameter and ‘b’ is the perpendicular diameter to ‘a’. One-Way ANOVA corrected for multiple comparisons (Holm-Sidak) was used to compare more than two experimental groups (Prism; GraphPad Software). Error bars represent standard error of the mean (SEM).

## SUPPLEMENTARY MATERIAL


