# Supplementary material for: Integrated in vivo genetic and pharmacologic screening identifies co-inhibition of EGRF and ROCK as a potential treatment regimen for triple-negative breast cancer
Source: Oncotarget. 2016 Jun 22;7(28):42859–72. doi: 10.18632/oncotarget.10230 (PMC5189992; doi:10.18632/oncotarget.10230)
Supplement: Supplementary file 1 [file oncotarget-07-42859-s001.pdf]

Integrated *in vivo* genetic and pharmacologic screening identifies co-inhibition of EGRF and ROCK as a potential treatment regimen for triple-negative breast cancer

Supplementary Material

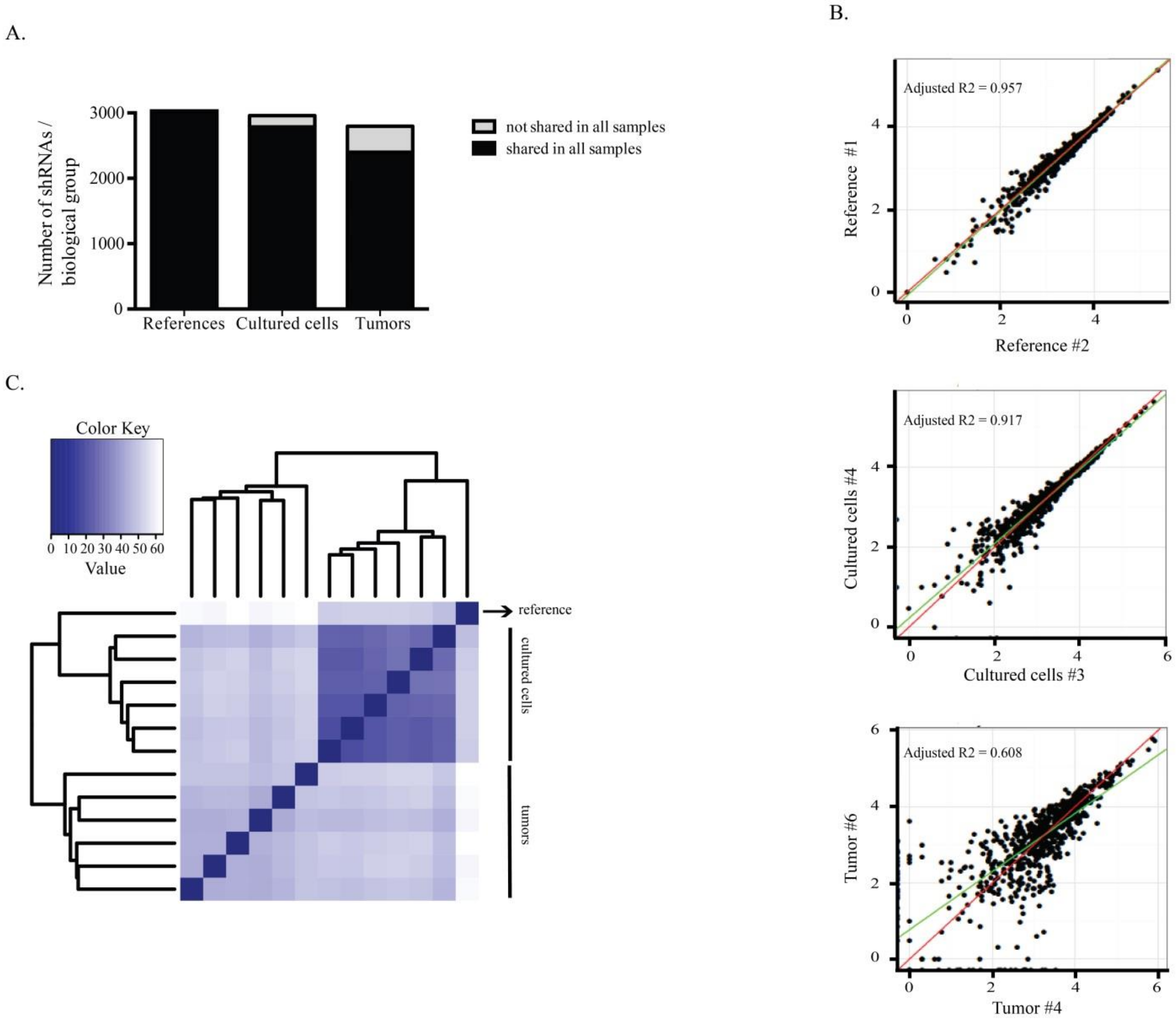

Supplementary Figure 1. A. The complexity of the library was retained in all groups in the MDA-MB-231 screen. Bars show the average number of shRNAs per biological group. Of the 3124 shRNAs detected in the reference samples, 2960 and 2795 were also found in cultured cells and tumors, respectively. Dark parts of the bars represent the shared shRNAs among the biological replicates within a group. 95% of the shRNAs were commonly found among the cultured cells while 89% were common among the tumors. **B.** Biological replicates correlated well with each other. A representative example from each sample group is shown. Every dot represents an shRNA. X- and y-axis show the abundance of shRNAs. **C.** Euclidean distance

heatmap showing the degree of similarity between all samples. All biological replicates in a sample group cluster together.

A.

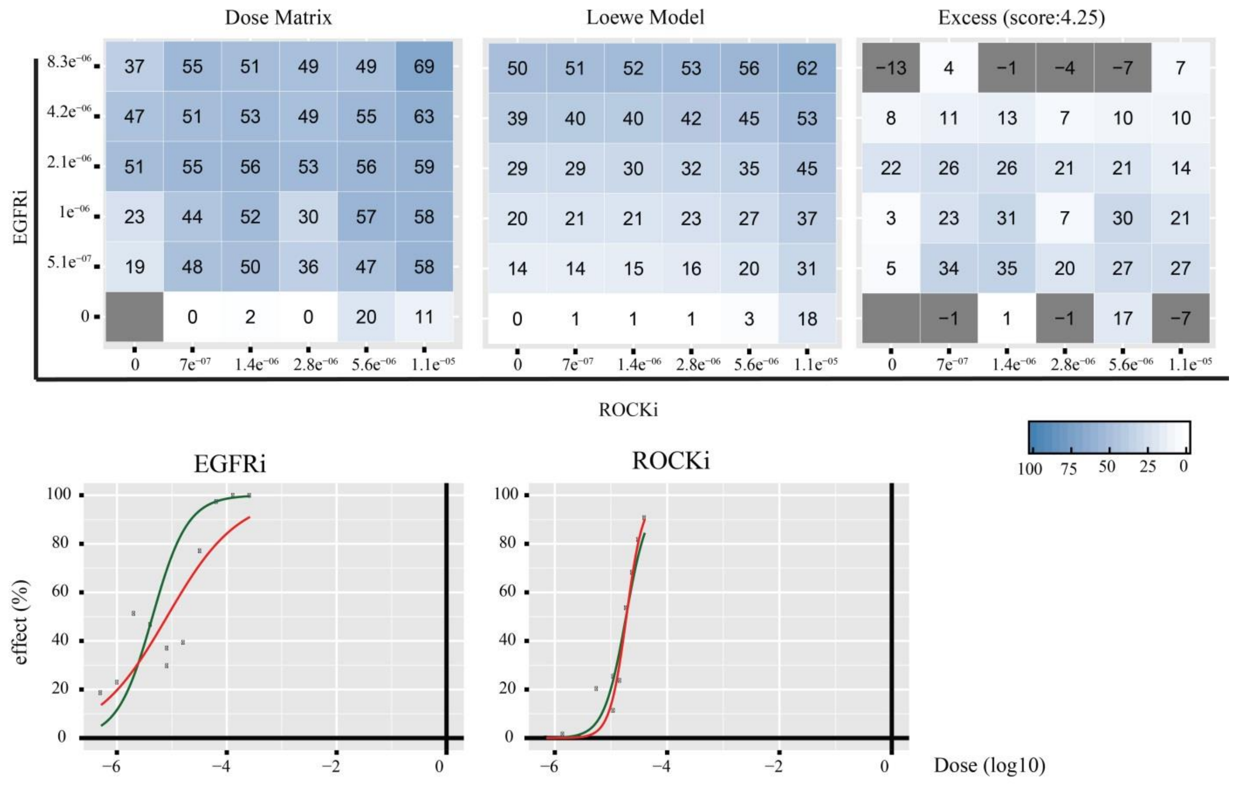
Dose Matrix
Loewe Model
Excess (score:4.25)
EGFRi
8.3e−06
4.2e−06
2.1e−06
1e−06
5.1e−07
0
37 55 51 49 49 69
47 51 53 49 55 63
51 55 56 53 56 59
23 44 52 30 57 58
19 48 50 36 47 58
0 2 0 20 11
50 51 52 53 56 62
39 40 40 42 45 53
29 29 30 32 35 45
20 21 21 23 27 37
14 14 15 16 20 31
0 1 1 1 3 18
−13 4 −1 −4 −7 7
8 11 13 7 10 10
22 26 26 21 21 14
3 23 31 7 30 21
5 34 35 20 27 27
−1 1 −1 17 −7
0 7e−07 1.4e−06 2.8e−06 5.6e−06 1.1e−05
ROCKi
100 75 50 25 0
EGFRi
ROCKi
effect (%)
100
80
60
40
20
0
−6 −4 −2 0
Dose (log10)

B.

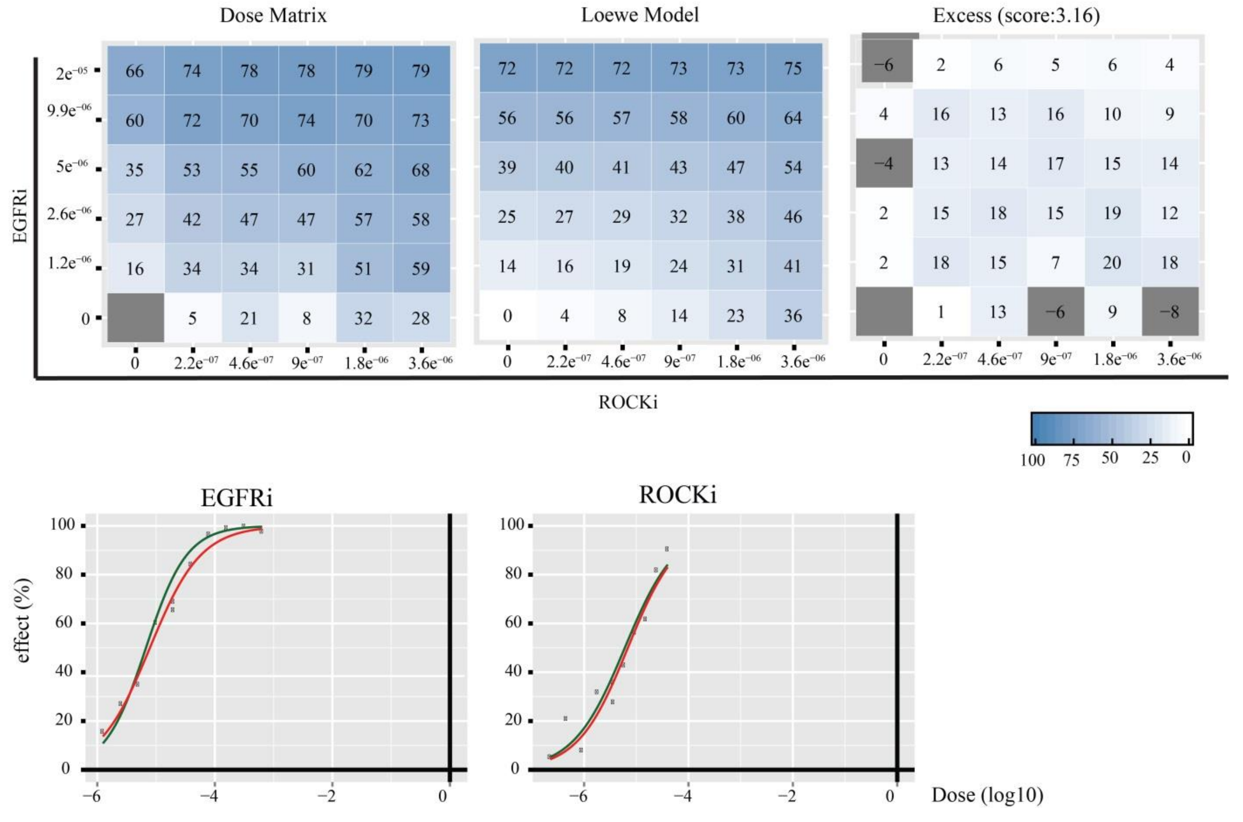
Dose Matrix
Loewe Model
Excess (score:3.16)
EGFRi
2e−05
9.9e−06
5e−06
2.6e−06
1.2e−06
0
66 74 78 78 79 79
60 72 70 74 70 73
35 53 55 60 62 68
27 42 47 47 57 58
16 34 34 31 51 59
5 21 8 32 28
72 72 72 73 73 75
56 56 57 58 60 64
39 40 41 43 47 54
25 27 29 32 38 46
14 16 19 24 31 41
0 4 8 14 23 36
−6 2 6 5 6 4
4 16 13 16 10 9
−4 13 14 17 15 14
2 15 18 15 19 12
2 18 15 7 20 18
1 13 −6 9 −8
0 2.2e−07 4.6e−07 9e−07 1.8e−06 3.6e−06
ROCKi
100 75 50 25 0
EGFRi
ROCKi
effect (%)
100
80
60
40
20
0
−6 −4 −2 0
Dose (log10)

Supplementary Figure 2. Synergy matrix results for the EGFRi and ROCKi combination in **A.** HCC1806 and **B.** MDA-MB-231 cells as a representative example. Dose Matrix shows the effect size of each combined dose upon treatment. Loewe Model shows the expected effect size of each combined dose based on the dose-response curves obtained within the same experiment. Subtraction of Loewe Model matrix from the Dose Matrix results in the Excess Matrix, from which a synergy score is derived.

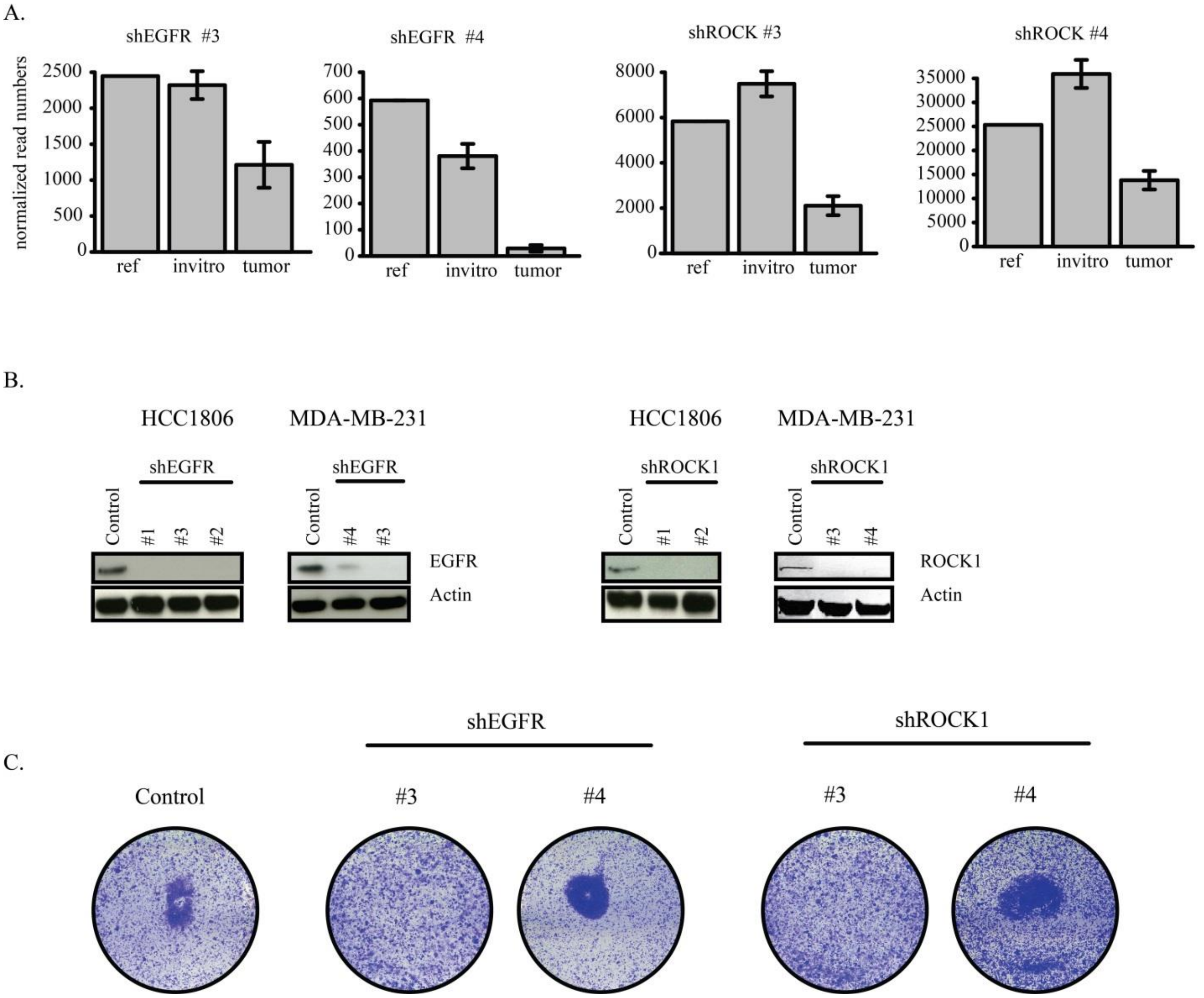

Supplementary Figure 3. A. shRNA read counts of the hairpins in the reference, cultured cells (invitro) and tumor samples as found in the MDA-MB-231 screen. **B.** HCC1806 and MDA-MB-231 cells were transduced with control shRNA and shRNA constructs against EGFR and ROCK identified as hits in the screen. Western blots show that all of these shRNAs efficiently downregulate their targets. **C.** MDA-MB-231 cells harboring shRNAs against EGFR and ROCK

were seeded on 6 well plates at $0.3*10^5$ cells/well. Cells were stained with crystal violet after six days. Corresponding normalized read numbers of each shRNA found in the screen is shown below the wells.

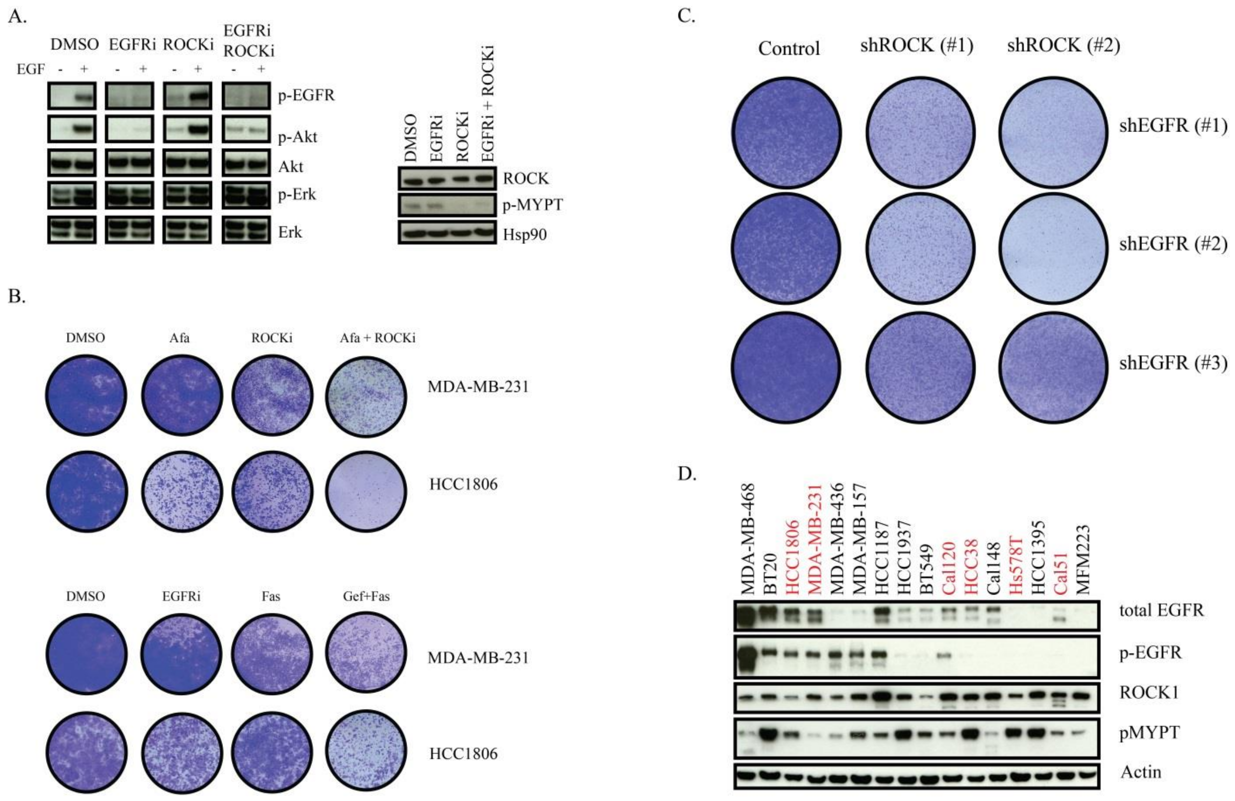

Supplementary Figure 4. A. MDA-MB-231 cells were treated with DMSO, EGFRi, ROCKi or EGFRi+ROCKi for two hours and stimulated with 30ng/ml EGF for 30 minutes. Western blots showing the changes in phosphorylated EFGR, Akt, Erk and MYPT levels. Hsp90 is used as loading control.. **B.** Other ROCK (Fasudil) and EGFR (Afatinib) inhibitors also show enhanced killing when combined with EGFRi or ROCKi, respectively. MDA-MB-231 cells were treated with 20μM EGFRi, 30μM Fasudil (Fas) or EGFRi+Fas and 1μM Afatinib (Afa), 4.8μM ROCKi, or Afa+ROCKi. HCC1806 cells were treated with 4.2μM EGFRi, 20μM Fas, or EGFRi+Fas and 1μM Afa, 2.4μM ROCKi or Afa+ROCKi. **C.** HCC1806 cells were transduced with control or shEGFR lentivirus constructs. After puromycin selection and expansion, $0.5*10^5$ cells were seeded on 6-well plates. The next day cells were transduced with control shROCK lentivirus constructs. Cells were stained with crystal violet fours days post-transduction. **D.** Western blot showing EGFR, phospho-EGFR, ROCK and phospho-MYPT levels of a panel of TNBC cell lines. Samples were collected after overnight serum-starvation and 30 minutes of EGF stimulation (30ng/ml).

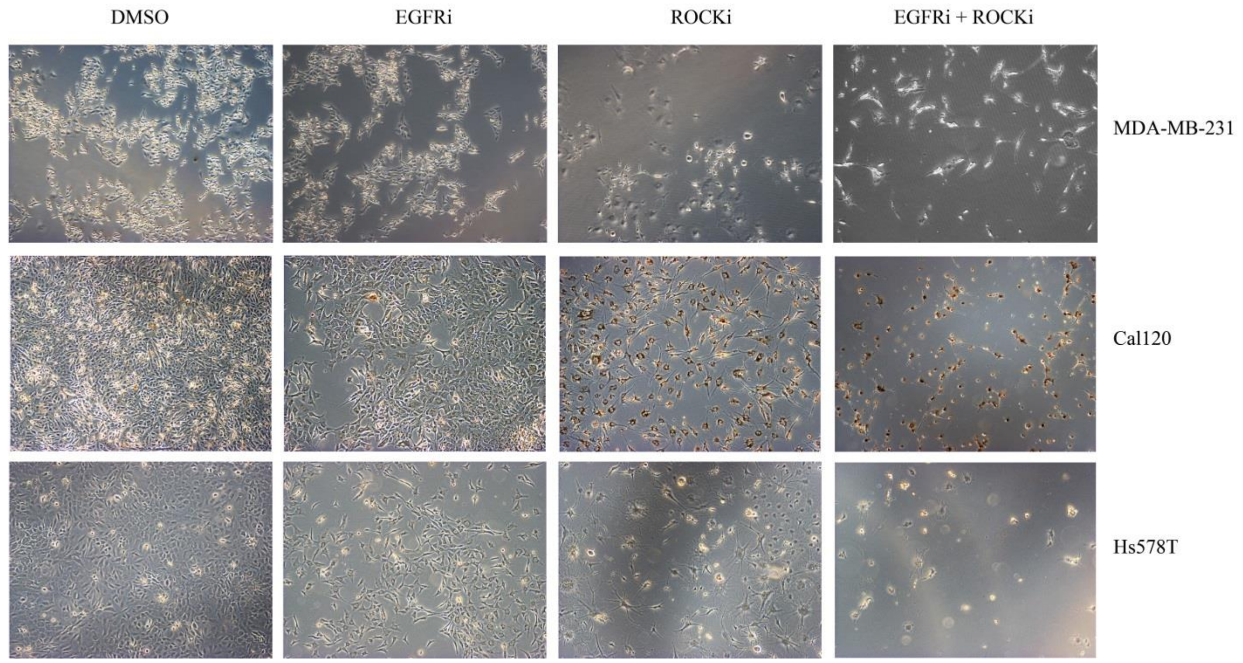

Supplementary Figure 5. Phase-contrast microscopy pictures of MDA-MB-231, Cal120, Hs578T upon five days of DMSO, EGFRi, ROCKi or EGFRi+ROCKi treatment (4X magnification).
